# Podocyte number and density changes during early human life

**DOI:** 10.1007/s00467-016-3564-5

**Published:** 2016-12-27

**Authors:** Masao Kikuchi, Larysa Wickman, Raja Rabah, Roger C. Wiggins

**Affiliations:** 1 Department of Internal Medicine, Nephrology Division, University of Michigan, 1570B MSRBII, 1150 W Medical Center Drive, Ann Arbor, MI 48109-0676 USA; 2 Faculty of Medicine, University of Miyazaki, Miyazaki, Japan; 3 Department of Pediatrics and Communicable Diseases, University of Michigan, Ann Arbor, MI USA; 4 Department of Pathology, University of Michigan, Ann Arbor, MI USA

**Keywords:** Podocyte, Glomerular maturation, Glomerular volume, Podocyte density, Glomerulosclerosis

## Abstract

**Background:**

Podocyte depletion, which drives progressive glomerulosclerosis in glomerular diseases, is caused by a reduction in podocyte number, size or function in the context of increasing glomerular volume.

**Methods:**

Kidneys obtained at autopsy from premature and mature infants who died in the first year of life (*n* = 24) were used to measure podometric parameters for comparison with previously reported data from older kidneys.

**Results:**

Glomerular volume increased 4.6-fold from 0.13 ± 0.07 μm^3^ x10^6^ in the pre-capillary loop stage, through 0.35 μm^3^ x10^6^ at the capillary loop, to 0.60 μm^3^ x10^6^ at the mature glomerular stage. Podocyte number per glomerulus increased from 326 ± 154 per glomerulus at the pre-capillary loop stage to 584 ± 131 per glomerulus at the capillary loop stage of glomerular development to reach a value of 589 ± 166 per glomerulus in mature glomeruli. Thus, the major podocyte number increase occurs in the early stages of glomerular development, in contradistinction to glomerular volume increase, which continues after birth in association with body growth.

**Conclusions:**

As glomeruli continue to enlarge, podocyte density (number per volume) rapidly decreases, requiring a parallel rapid increase in podocyte size that allows podocyte foot processes to maintain complete coverage of the filtration surface area. Hypertrophic stresses on the glomerulus and podocyte during development and early rapid growth periods of life are therefore likely to play significant roles in determining how and when defects in podocyte structure and function due to genetic variants become clinically manifest. Therapeutic strategies aimed at minimizing mismatch between these factors may prove clinically useful.

## Introduction

Podocytes are highly differentiated post-mitotic neuron-like cells with a limited capacity for replacement [1–5]. The realization that glomerular failure can be initiated and driven in a podocyte-dependent manner by glomerular enlargement per se, in the absence of immune, inflammatory or other insults to the glomerulus, provides insight into potential mechanisms of the progression of glomerular diseases [6, 7]. This concept is further amplified by recent studies on glomerular aging in which progressively decreasing podocyte density (number per glomerular tuft volume) with age is associated with hypertrophic podocyte stress and mass podocyte detachment, glomerular tuft collapse, and focal global glomerulosclerosis [8]. All progressive glomerular diseases are associated with an increased rate of podocyte detachment [9]. Thus, all progressive glomerular diseases that have an impact on adults can be seen to be superimposed upon a steadily declining podocyte density with age, such that older people are more severely affected than younger people, whatever the underlying glomerular insult [8]. These concepts can help to explain the over-riding impact of older age as a dominant risk factor for both chronic kidney disease and end-stage kidney disease (ESKD) [10]. They also offer new approaches for monitoring progression and response to treatment in glomerular diseases [11].

At the other end of the age spectrum a different constellation of glomerular diseases predominates, particularly affecting children and young adults, in which podocyte depletion and dysfunction also play key roles [5]. In one report, 85% of steroid-resistant nephrotic syndrome (SRNS) cases by 3 months of age and 66% of cases with onset by 1 year could be explained by recessive mutations in one of four genes (*NPHS1*, *NPHS2*, *LAMB2* or *WT1*) [12]. To date, more than 40 genes have been identified as causing glomerular phenotypes, with some genes expressed specifically by podocytes (e.g., *NPHS1* and *NPHS2*), whereas others are quite widely expressed, but, for unexplained reasons, present with a glomerular phenotype [12].

A spectrum of glomerular phenotypes can be identified and linked to podocyte dysfunction [5]. These include diffuse mesangial sclerosis (DMS), representing failed glomerular development at the pre-capillary loop stage (e.g., *PLCE1*) [13, 14]; congenital nephrotic syndrome (CNS), representing failed glomerular development at the capillary loops stage caused by variants in podocyte-expressed genes required for foot process structure and function (e.g., *NPHS1*, *NPHS2*) [5, 12, 15, 16]; minimal change nephrotic syndrome (MCD) manifests by diffuse foot process effacement and podocyte dysfunction that particularly affects children in the first years of life and is steroid-sensitive (e.g., *EMP2*, *MAGI2*, *TENC1*, *DLC1*, *CDK20*, and *ITSN1*) [12, 17]; and focal and segmental glomerulosclerosis (FSGS), representing podocyte depletion and dysfunction with characteristic segmental scarring occurring after glomerular development is completed [5]. More than 40 gene variants have been shown to confer susceptibility to FSGS, including genes that are also associated with DMS, MCD, and CNS phenotypes [12]. In addition, defects in collagen IV α3, 4, and 5 (*COL4A3*, *COL4A4*, and *COL4A5*) genes traditionally causing the Alport syndrome complex are now recognized to also cause an FSGS phenotype associated with podocyte depletion [18–21], and *ApoL1* gene variants prevalent in African–Americans are a major cause of FSGS phenotypes [22, 23]. These clinical syndromes do not have major immune or inflammatory components, suggesting that they too, may, in part, be related to hypertrophic processes active in the perinatal and active growth periods of life. FSGS associated with obesity, large body size, and acromegaly are also examples where FSGS development appears to be directly linked to growth [6]. This is important conceptually, because if the translation from genetic susceptibility to clinical phenotype is caused in part by hypertrophy-induced events, then therapeutic strategies could potentially be directed against hypertrophy and related factors that tend to amplify the mismatch between glomerular volume and podocyte mass.

To examine this question we performed podometric studies to map the changes experienced by podocytes during development and early life. The data show that large increases in glomerular volume mandate parallel changes in podocyte density and cell size, thereby imposing major hypertrophic demands that have to be accommodated if podocytes are to successfully navigate the early years of life. These hypertrophic forces likely play a role in determining how genetic susceptibilities are transduced into clinical phenotypes and how fast they drive progression toward ESKD.

## Materials and methods

### Validation of the cohort

To ensure that the characteristics of the cohort were comparable with normal ranges, the body weight at birth was compared with normal reported ranges for fetal development [24] and post-natal growth was compared with average body weight for boys and girls using weight-for-age charts available through the National Center for Health Statistics [25]. For calculation of the normal post-conceptional weight range from these tables, we assumed that normal gestation length was 280 days [26].

### Podometric methodology

The method used was as previously reported [27, 28]. In brief, formalin-fixed kidney tissues from autopsy were sectioned from paraffin blocks at 3 μm. Podocytes were identified if they expressed both GLEPP1, a podocyte-specific protein tyrosine phosphatase, and TLE4, a transcription factor expressed robustly by podocyte nuclei and co-localizing with WT1, as previously described [27, 28]. TLE4 immunofluorescence with triple amplification was performed for podocyte nuclei and GLEPP1 immuno-peroxidase staining was performed for podocyte cytoplasm, as previously described [27, 28]. Imaging of immunofluorescence was performed by photographing glomeruli in the red, green, and blue channels and creating composite images in which red fluorescence represented TLE4 and green fluorescence represented nonspecific fluorescence to allow structures that fluoresced nonspecifically to be recognized and eliminated from consideration. Composite images including blue DAPI staining were used to identify nuclei so that in each composite image all red TLE4-containing structures could be confirmed to be nuclei. Podometric parameters were calculated by using the previously elucidated quadratic equation [27] that employs:

1. The observed number of podocyte nuclei transected per tuft cross-section
2. The mean podocyte nuclear caliper diameter derived using Image-Pro software
3. The histological section thickness
4. A podocyte nuclear shape coefficient
5. The tuft cross-sectional area
6. The percentage of the tuft area that is GLEPP1-positive

Glomerular volume was estimated according to Weibel and Gomez [29]. In each 3-μm-thick tissue section, >20 consecutive glomeruli were sampled systematically across the section and back so that an averaged sample representative of all glomeruli present was obtained. As the blocks of autopsy kidney tissue used had not been cut in a systematic way in relation to kidney structural orientation, we were not able to differentially evaluate superficial and deep glomeruli.

### Integration of perinatal and later age podometric data

Data from the current study were integrated with previously reported adult podometric data [8]. For this analysis 38-week post-conceptional age is defined as 0 days postnatal age. The postnatal age of patients with a conceptional age of less than 38 weeks was represented as a negative number.

### Statistical methods

For descriptive purposes, the mean ± SD was used to show the distributions of continuous variables. A linear regression model was utilized to quantify the relationship between the continuous variables. ANOVA was used for comparisons among multiple groups using Bonferroni correction for multiple comparisons. The level of significance was accepted at *p* <0.05. Analyses were performed using SPSS software, version 21 (IBM, Armonk, NY, USA). One data set was excluded from analysis because the glomerular volume was more than twice that of any other sample (a statistical outlier). The patient had trisomy 21 with ovarian and pulmonary hypoplasia and esophageal atresia. Histological evaluation of the kidney showed fewer large glomeruli, which was suggestive of nephronopenia with compensatory glomerular enlargement.

## Results

Table 1 shows group demographic data from 24 autopsied children who died from a variety of causes at age <1 year without kidney disease being the proximate cause of death. Most (72%) were pre-term deliveries, defined as birth before 38 weeks’ gestation.

**Table 1:** Demographics of the study population (*n* = 24). Race was identified in 10 cases (6 Caucasian [60%], 3 African–American [30%], and 1 Hispanic [10%])

	Mean ± 1SD	Range
Age post-delivery (days)	47 ± 61	0–240
Gestational age (weeks)	32 ± 6	18–40
Male:female	16:8	
Birth weight (g)	1,917 ± 1,165	205–4,000
Body weight at autopsy (g)	3,204 ± 2,612	205–10,000
Combined kidney weight (g)	29 ± 23	2.5–92.9
Cause of death (%)		
Cardiac failure	33	
Respiratory failure	38	
Infection	21	
Extreme prematurity	13	
Other	29	

**Fig. 1:**
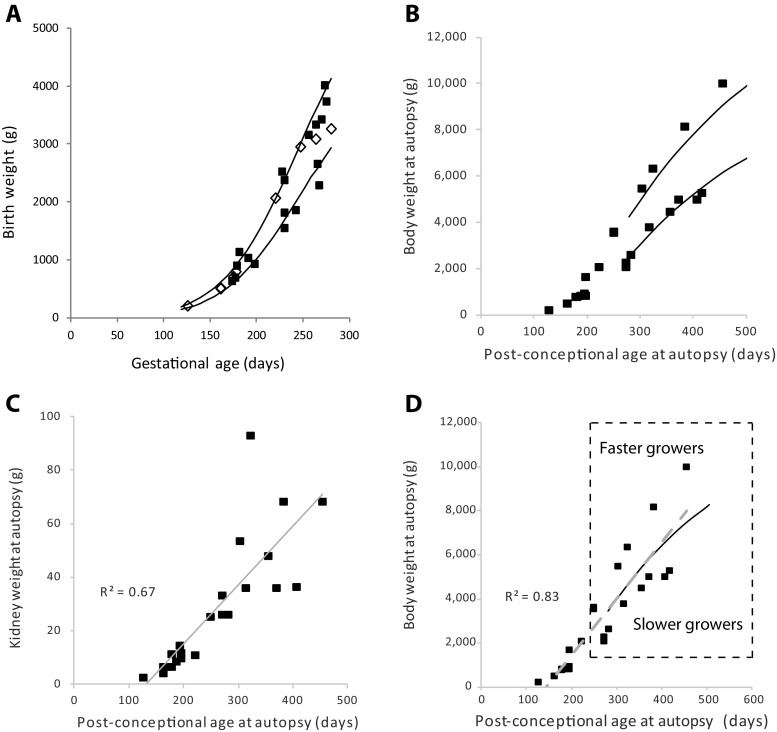
Relationship of body weight to gestational and post-gestational ages. **a** Birth weight in relation to gestational age. The *solid lines* show the 10th and 90th percentiles for normal intrauterine growth. The cohort conformed to expected values for intrauterine growth. Those that survived <1 week are shown as *open diamonds*. Those that survived >1 week are shown by *closed squares*. There was no differences in the rate of intrauterine growth between those who died soon after birth and those who survived longer. **b** Body weight at death in relation to post-conceptional age. The 5th and 95th percentile range for the average value for boys and girls as shown by CDC growth charts is shown by the *solid lines* (assuming that normal gestation lasts 280 days). The cohort growth rates conformed approximately to the expected ranges. **c** Relationship between body weight and kidney weight at autopsy. Kidney weight was linearly related to body weight over the time period. **d** Faster growers versus slower growers. The plot is the same as in Figure 1b above. The *solid line* shows the 50th percentile growth curve for average boys and girls. The *gray long-dashed line* shows the correlation line between body weight and post-conceptional age for the cohort as a whole (R^2^ = 0.83). These lines overlap, suggesting that on average the cohort represents an approximation of normal growth. To extend this analysis to determine to what effect slower versus faster growth would have on podometric measurements we compared a group that fell above the 50th percentile (“faster growers” [*n* = 6]) with a group that fell below the 50th percentile (“slower growers” [*n* = 8]) as shown by the box delineated by the *dashed line*. The comparison data are shown in Table 2. No statistically significant differences in podometric parameters were observed between the “faster” and “slower” growing groups, suggesting that differences in the growth rate within the range observed would not have a large impact on podometric parameters, although this would affect kidney weight

One concern with using autopsy samples for analysis is that the severity of illness culminating in death would itself disrupt normal kidney development so severely that podometric data measured in these samples would not be representative of the normal state. To address this question, we first analyzed the cohort to determine whether or not growth data distributed within expected norms for gestational and post-conceptional age. Figure 1a shows how body weight at birth was related to gestational age at birth for the cohort as a whole. The 10th and 90th centile ranges overlap with the observed cohort data, showing that the cohort had relatively normal intrauterine growth. Furthermore, there were no differences in intrauterine growth rate between those who died soon after birth and those who survived longer suggesting that those who died shortly after birth had grown relatively normally in utero. Figure 1b shows how body weight continued to increase after birth in relation to the normal 5th and 95th percentile ranges. Figure 1c shows how kidney weight was linearly related to post-conceptional age. Figure 1d shows a further analysis of the data shown in Fig. 1b. The 50th percentile averaged growth for boys and girls overlaps with the correlation line for the cohort as a whole, suggesting that average growth in the cohort might have approximated expected values. To further examine the effect of faster or slower growth, we compared a group whose weight gain was above the 50th percentile range (“faster growers”) and a group whose rate of weight gain was below the 50th percentile range (“slower growers”). Table 2 compares body weight, kidney weight, glomerular volume, podocyte number per glomerulus, and podocyte density between these two groups to determine whether growth rate differences of the degree observed would significantly affect podometric parameters. The “faster grower” group had significantly higher body weight (*p* = 0.04) and kidney weight (*p* = 0.02); however, none of the podometric parameters were significantly different in the “faster” and “slower” growing groups. We therefore conclude that whether or not delivery occurred prematurely (before 38 weeks), or death occurred earlier or later, or weight gain for age was above or below the 50th percentile, the relationships of podometric parameters with post-conceptional age represented a continuum that was not impacted in a major way by the inter-current illness causing death. Therefore, podometric data from the cohort could reasonably be interpreted as being representative of early human life.

**Table 2:** Comparison of faster growers with slower growers

	*n*	Body weight at birth (g)	Body weight at autopsy (g)	Kidney weight (g)	Glomerular volume (×10^6^ μm^3^)	Podocytes per glomerulus (*n*)	Podocyte density (per 10^6^ μm^3^)
Slower growers	8	2,666 ± 840	3,820 ± 1,328	34.5 ± 7.5	0.60 ± 0.20	613 ± 188	1,054 ± 217
Faster growers	6	2,515 ± 1,089	6,201 ± 2,545	61.5 ± 24.9	0.50 ± 0.36	521 ± 105	1,169 ± 344
*p* value		0.78	0.04	0.02	0.36	0.30	0.46

One case in the original cohort was identified where the glomerular volume was more than twice that of any other case. This case was a statistical outlier (>2 standard deviations above the mean) and associated with both ovarian and pulmonary hypoplasia and possible renal hypoplasia with small kidneys at autopsy. This case was excluded from the cohort used for further analysis as outlined in the Materials and methods. There were no significant differences noted between the sexes (16 males and 8 females). Race was not known in 15 out of 24 cases (63%); thus, no inferences related to race can be made from this study.

**Fig. 2:**
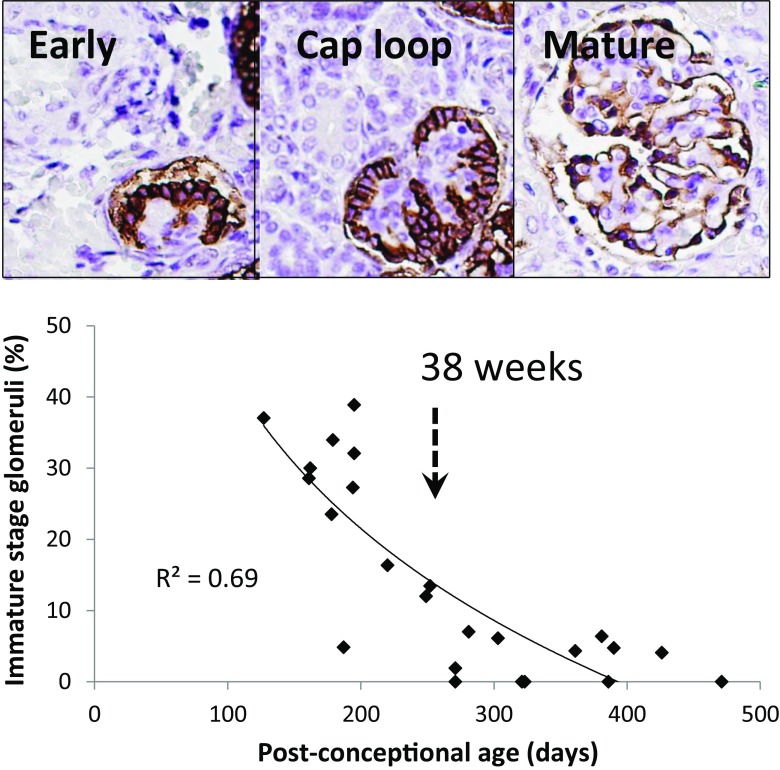
Relationship of glomerular maturity to gestational age. *Upper panel*: GLEPP1 immuno-peroxidase was used to map the stages of glomerular development. All pre-capillary loop stage glomeruli were designated as “immature” development. All capillary loop stage and mature glomeruli were designated as “mature” development. *Lower panel*: the relationship of stage of development (as defined in the text) to gestational age. By 38 weeks’ gestation <10% of glomeruli were scored as at an “immature” stage of development

In humans, nephrogenesis begins by 9 weeks and is thought to be completed between 32 and 36 weeks’ gestation [30]. Glomerular development was divided into groups defined as “immature” (pre-capillary loop stage) and mature (post-capillary loop and mature stage), as shown in Fig. 2. The proportion of glomeruli at “immature” stage development in relation to post-conceptional age is also shown in Fig. 2. By a post-conceptional age of 38 weeks <10% of glomeruli were at “immature” stage development. The proportion of immature glomeruli at any point in time is therefore related to post-conceptional age, whether inside or outside the uterus.

**Fig. 3:**
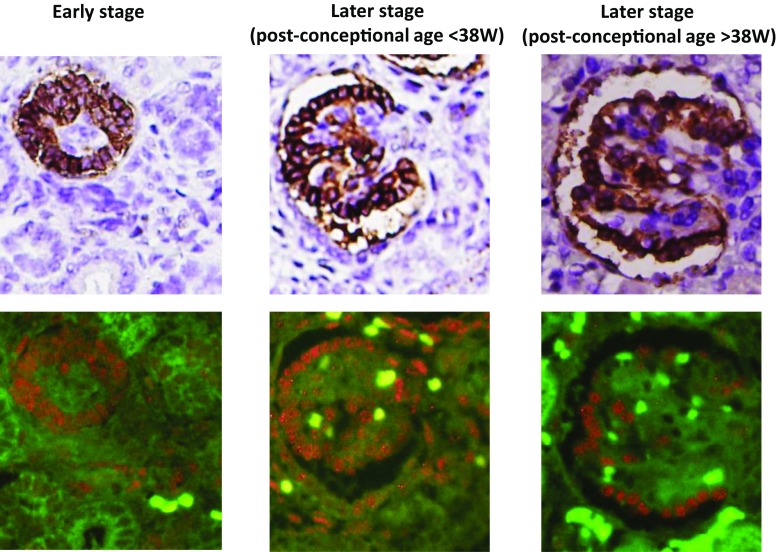
Podometric estimations. Representative glomeruli at an “Immature stage,” mature stage (<38 weeks’ gestation) and mature stage (≥38 weeks’ gestation) are shown by GLEPP1 peroxidase staining (*upper panels*) and TLE4 red immunofluorescence (*lower panels*). Podocytes were identified by their having both GLEPP1-positive cytoplasm and TLE4-positive nuclei. Nonspecific TLE4 positive staining was observed in the interstitial compartment in some cases. Podocyte nuclear number, podocyte nuclear mean caliper diameter, GLEPP1-positive area and total glomerular tuft area were measured using software, as outlined in Materials and methods

To understand the time course of podometric changes with development we divided glomeruli into three groups. These included “immature” (pre-capillary loop), “mature (post-capillary loop) at less than 38 weeks’ gestation (<38 weeks)”, and “mature (post-capillary loop) at 38 weeks or greater of gestation (≥38 weeks)” (Fig. 3). The podometric methodology used requires the glomerular structure to be approximately spherical to estimate podocyte parameters; thus, the earliest stages of glomerular development, which are nonspherical, were not evaluated.

**Fig. 4:**
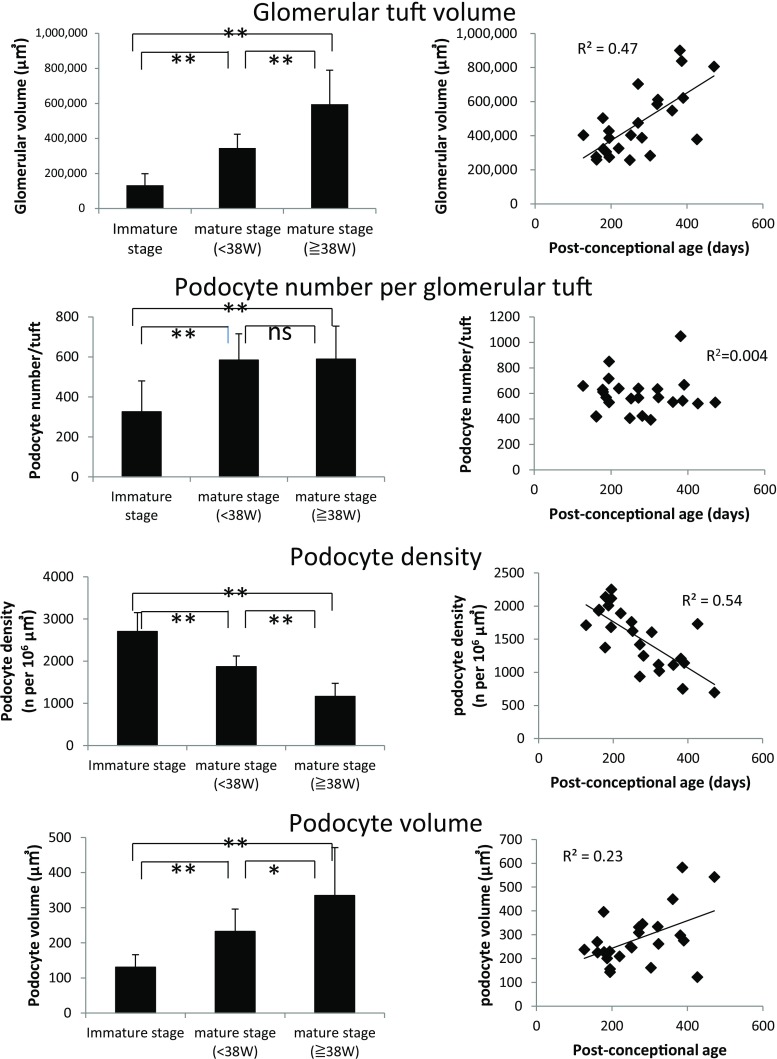
Changes in podometric parameters occurring with glomerular development and post-conceptional age. *Left panels* show a comparison of three stages of glomerular development including immature development, mature <38 weeks’ gestation stage and mature ≥38 weeks’ gestation stages. The *right panels* show individual data points for all mature stage glomeruli in relation to post-conceptional age. These data emphasize how glomerular volume increases as post-conceptional age increases and as glomerular development proceeds. In contrast, podocyte number increases from the immature stage to the capillary loop stage to reach a maximum averaging about 600 podocytes per tuft, but does not increase thereafter in association with continuing glomerular enlargement. Podocyte density, therefore, decreases with development and post-conceptional age requiring podocyte size to increase 1.8-fold between immature and mature (<38 weeks) and 2.6-fold by mature (≥38 weeks’ gestation). Values are mean ± SD. *p<0.05, **p < 0.01

As shown in Table 3 and Fig. 4, glomerular volume increased 2.7-fold from the “immature stage” to the “mature stage at <38 weeks” and 4.6-fold from the “immature stage” to the “mature stage at ≥38 weeks’” gestation. Podocyte nuclear number per glomerulus increased from 326 ± 154 in the “immature stage” glomeruli to 584 ± 131 in the “mature stage at <38 weeks” glomeruli, but did not further increase significantly between “mature stage development at <38 weeks” and “mature stage at ≥38 weeks” (589 ± 166 podocytes per glomerulus). Therefore, by the capillary loop stage of glomerular development, glomeruli had acquired their full complement of podocytes, although podocytes remained small and tightly packed at high density into small glomeruli. As development proceeded, podocyte density decreased in association with glomerular enlargement, such that the (estimated) podocyte volume was required to increase approximately 1.8-fold between the “immature stage” and the “mature stage at <38 weeks” and 2.6-fold by the “mature stage at ≥38 weeks”. Individual data points for this analysis are also shown in Fig. 4. These data emphasize the large changes in podocyte density and estimated cell volume that occur over the perinatal period in humans.

**Table 3:** Podometric values for stages of glomerular development

	Immature stage	Mature stage <38 weeks	Mature stage ≥38 weeks
Glomerular volume (μm^3^ x 10^6^)	0.13 ± 0.07	0.35 ± 0.08	0.60 ± 0.19
	(0.03–0.24)	(0.26–0.50)	(0.28–0.90)
Podocyte number per glomerulus	326 ± 154	584 ± 131	589 ± 166
	(71–549)	(406-850)	(393–1048)
Podocyte density (per 10^6^ μm^3^)	2,702 ± 451	1,870 ± 250	1,166 ± 310
	(1,731–3,649)	(1,375-2,253)	(696–1732)
Total podocyte volume (x10^3^ μm^3^)	41.5 ± 23.4	136.0 ± 45.3	195.1 ± 81.7
	(8.2–80.8)	(86.8–260.0	(64.1–320.8)
Average podocyte volume (μm^3^)	131 ± 36	233 ± 64	335 ± 136
	(76–215)	(143–397)	(122–583)

“Immature stage” of development is at the pre-capillary loop stage. “Mature <38 weeks” includes all glomeruli at the capillary loop and later stages of development in kidneys before 38 weeks’ gestation. “Mature stage >38 weeks” includes all glomeruli at the capillary loop and later stages of development in kidneys at ≥38 weeks’ gestation. Values are mean ± SD with the range shown in parentheses. Total podocyte volume is the GLEPP1-positive area multiplied by the glomerular volume to give an estimate for the average volume of all podocytes present in glomeruli

**Fig. 5:**
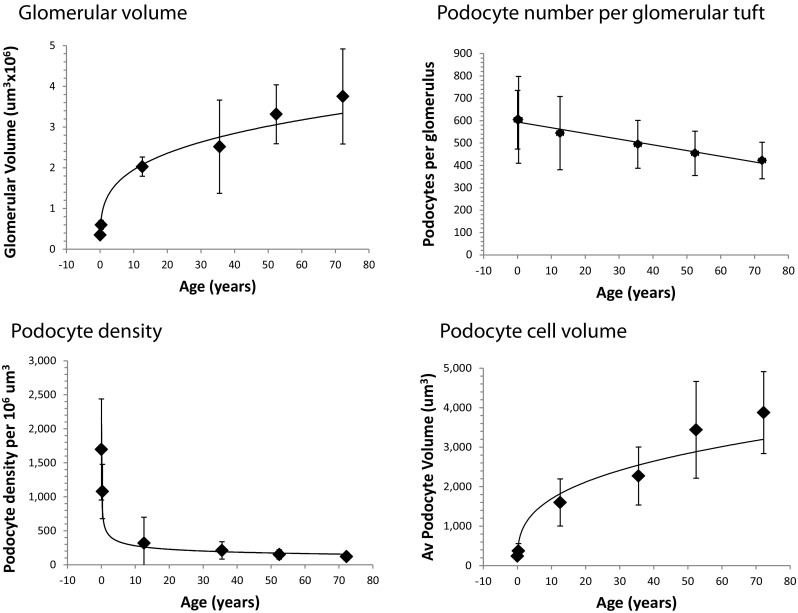
Changing podometric parameters throughout life. Podometric parameters for more mature glomeruli at <38 weeks and >38 weeks post-conception are shown in relation to previously published podometric parameters at 12.6, 35.5, 52.2, and 72.2 years of age. Glomerular volume can be seen to rapidly increase during the early growing stage of life and to continue to increase at a slower rate at later stages of adulthood. Podocyte number per tuft starts at about 600 and steadily decreases throughout life at a rate of 0.5% per year. Because of the very rapid increase in glomerular volume occurring during periods of rapid growth in childhood and adolescence, podocyte density decreases very rapidly, requiring a proportionate rapid increase in podocyte cell size. The continued glomerular volume increase and podocyte number decrease throughout adulthood mean that podocyte density decreases and requires podocyte volume to increase proportionately to cover the filtration surface area [7]

To put these perinatal podometric data into context, Fig. 5 compares the “mature stage” glomeruli at both <38 weeks and ≥38 weeks with glomeruli at other phases of life, using previously reported data [8]. During the perinatal period and over the first 12 years of life glomerular volume can be seen to increase about 5-fold, whereas podocyte number per glomerulus begins the steady decline that will continue over the life of the kidney. These changes mandate a large reduction in podocyte density over time that in turn requires the podocyte to increase its volume, estimated to be approximately 7-fold by 12 years and 17-fold by 72 years of age.

## Discussion

To maintain the normal filtration barrier, the podocyte must completely cover the filtration surface area with foot processes. At the same time, our data show that the podocyte nuclear number per glomerulus does not increase after the capillary loop stage of glomerular development, in spite of continued glomerular enlargement that rapidly occurs in the perinatal and postnatal periods, and at a lesser rate throughout life. Some reports have suggested that podocyte replacement may occur from the parietal and/or juxta-glomerular compartments [31–36]. However, the quantitative capacity of these replacement pathways and the time period during development and post-natal life at which they may occur remain poorly defined, and quantitative studies suggest that their capacity for podocyte replacement might be small [37]. Significantly, podocytes are therefore required to cope with the demands of an increasing filtration surface area by hypertrophy. We previously modelled this process and demonstrated that failure of the podocyte per se to cope with hypertrophic stress results in proteinuria, adhesions to Bowman’s capsule, and development of glomerulosclerosis culminating in ESKD [6]. Thus, podocyte hypertrophic stress in its own right can be a driver of progression to ESKD [6, 7].

Podocyte cell structure is complex, consisting of a cell body and major and intermediate processes that serve as a platform for tertiary (actin-based) foot process attachment to the underlying basement membrane, allowing inter-digitating foot processes between neighboring cells to form specialized intercellular junctions (slit diaphragms) through which the glomerular filtrate is formed. Foot processes use an integrin-based protein complex to attach and deliver key components to the underlying glomerular basement membrane (GBM) and thereby to communicate with endothelial and mesangial cells [38, 39]. Maintaining these critical foot process inter-relationships under hypertrophying conditions, particularly when rapidly occurring over short periods of time, must impose remarkable logistical challenges in space and time that have to be accommodated by as yet poorly understood signaling mechanisms.

With realization of the major developmental and hypertrophic stresses faced by the podocyte in the perinatal period, this cell is anticipated to be especially susceptible to genetic defects that might compromise its adaptive capacities. On the one hand, these include defects in specialized proteins uniquely expressed by podocytes and required for this cell to develop and perform its specialized functions, including forming and maintaining foot processes and slit diaphragms essential for filtration (e.g., *NPHS1* [nephrin] and *NPHS2* [podocin]) [15, 16] and delivery of the specialized type IV collagen alpha 3, 4, and 5 chains required to maintain the underlying mature GBM structure [38]. On the other hand, genetic variants in proteins common to many cell types are also associated with a proteinuria and nephrotic syndrome phenotype. Examples include signaling, sensing and coordinating proteins (e.g., PLCE1, TRPC6, ARHGAP24, and ARHGDIA), cytoskeletal proteins (e.g., alpha actinin 4), adhesion proteins by which podocyte foot processes attach to the underlying basement membrane (e.g., ITGA3, ITGA4, and LAMB2) and co-enzyme Q pathway synthesis proteins necessary to protect the huge lipid surface area of the podocyte from mitochondria-induced oxidant damage [12, 22, 40]. In other words, the hypertrophic and signaling stresses experienced at particular times during podocyte development may demand an optimized functional capacity for some molecular pathways that are less critical in other cell types, thereby potentially explaining why variants in these rather widely expressed proteins cause a proteinuria and nephrotic syndrome phenotype at particular ages and stages of glomerular development.

The methodology used for our study provides only an approximate estimation of podocyte volume because the complexity of the foot process structure is not fully taken into account by the peroxidase immuno-histochemical staining approach. Nevertheless, approximations can be conceptually useful. In this report, we estimate that in mature glomeruli of the developing kidney (<38 weeks’ gestation to >38 weeks of gestation), the average podocyte volume increases at a very rapid rate. By 13 years of age (adolescence) the rate of podocyte enlargement remains high, but is at only one third the perinatal rate. During adult life between 20 and 72 years of age podocytes are still increasing in volume, but at a much slower rate (about 10% of the perinatal rate). However, over a life-time this slower rate is associated with hypertrophic stress events associated with the development of global glomerulosclerosis [8].

A concern with the current study is that although body growth rates were quite well maintained up until time of death in the cohort examined, the kidney samples came from autopsies of patients who had died rather than being derived from biopsies obtained under normal physiological conditions. The events preceding death would likely have had an impact on the development of the kidney and other organs to some extent. Sutherland and colleagues demonstrated accelerated postnatal renal maturation following preterm birth by matching preterm infants and post-conceptional age-matched control infants who died suddenly in utero [41]. Furthermore, in a baboon model of preterm birth, Gubhaju and colleagues reported a marked increase in kidney size relative to control kidneys in the first 3 weeks after birth, in spite of no increase in body weight [42]. They suggested that this could represent a compensatory response to the increased functional demands placed on the preterm kidney as a result of separation from the maternal milieu. Mechanisms leading to accelerated renal maturation observed in the preterm kidney are not well understood, but may also include factors that promote organ maturation, such as exposure to glucocorticoids, which are commonly administered before preterm delivery to aid postnatal respiratory function. Similar events would have occurred in our study sample. Therefore, although we document relatively normal growth occurring in the cohort used, interpretation of the data provided in this report must recognize these caveats, which could have an impact on the detailed data, but will probably not change the overall conclusions.

The methodology used in this study has been validated by comparisons with other methods, including ultrastructural methods [27, 28]. However, as noted above, podocyte structure is highly complex, so that immuno-peroxidase at the light microscopic level cannot provide a complete picture of actual podocyte cell volume. Nevertheless, this histological approach is reproducible, usable in archival paraffin-embedded tissues available from autopsy and biopsy series, and provides an approximation of the changes in cell size occurring over time. Puelles and colleagues, using a different morphometric method, reported autopsied kidney values from 4 children (aged 0.25 to 3 years) and 12 adults (aged 25 to 49 years) [43]. They reported that glomeruli from 25- to 49-year-olds contained statistically more podocytes (558 per glomerulus) than children (452 per glomerulus), although the glomeruli from children were smaller and therefore their podocyte density was higher than in adult glomeruli, as we also observed. The values obtained, including podocyte number per glomerulus, were within the range of those observed in the current study, although our data showed that podocyte number per glomerulus reached a maximum plateau by the capillary loop stage of development and did not appear to increase thereafter. Crobe and colleagues reported that podocyte number decreased about 40% (from 1,908 to 1,126 per glomerulus) from fetus to full term [44]. The estimated number of podocytes per glomerulus in the Crobe report was 2- to 3-fold higher than our estimate and that of Puelles and colleagues [43]. These differences could well reflect the methodologies used, where podocytes in the Crobe report were identified at the light microscopic level without specific markers.

A further limitation of this study is that we focused on podocyte parameters, when in reality the glomerulus is a dynamic complex of interacting cell types (endothelial, mesangial, parietal epithelial, juxta-glomerular cells, and podocytes) and structures (GBM, mesangial matrix, Bowman’s capsule) perfused by pulsatile blood flow at high pressures. The observed podocyte density decrease during development occurs in association with complex events restructuring the glomerulus. These include, but are not limited to, the formation of new basement membrane, including switching collagen IV chains from α1 and α2 trimers to α3, 4, and 5 trimers made and secreted by podocytes into the GBM [37], extension and in-folding of capillary loops that increase the filtration surface area, increased blood space, and flow within capillary loops [38], formation of the mesangium and mesangial matrix, and accumulation of other glomerular cells, including endothelial, mesangial, juxta-glomerular, and parietal epithelial cells. Each of these processes requires specialized molecular machinery and regulation by signaling within and between cells. However, in spite of this complexity, there is now strong support for the concept that:

1. Podocyte depletion itself drives progression to ESKD
2. All genetic causes of glomerular phenotypes so far identified are in proteins expressed by podocytes
3. Progression in all glomerular diseases so far examined is associated with podocyte depletion [11]

Progress in science is often made by simplifying complex problems to make them accessible to the scientific method. The availability of specific markers that allows quantitation of podocyte parameters offers tools that can be used to reach into the glomerulus to dissect events [11]. Therefore, a focus on podocytes as a step toward trying to understand the biological complexities involved in glomerular diseases associated with early life is not unreasonable, although it should be kept firmly in mind that all changes observed in podocyte parameters will have their counterparts in other glomerular cell types and structures that together describe the complexity of glomerular diseases.

In summary, while underlying genetic “causes” of the nephrotic syndrome of childhood diseases are being identified in an increasing proportion of cases [45], the precise mechanisms by which these genetic variants become transduced into clinical syndromes remains largely undefined. The biology of the developing and growing glomerulus must be part of the explanation that links the underlying genetic susceptibilities and environmental factors (e.g., growth rate and weight gain) that combine to drive podocyte stress, leading to glomerular dysfunction and progressive glomerulosclerosis in a particular case. Understanding these stresses will provide insight into mechanisms and potentially offer strategies for mitigating the mismatch between hypertrophic demands and the podocyte’s capacity to comply.
